# Snapshot computational spectroscopy enabled by deep learning

**DOI:** 10.1515/nanoph-2024-0328

**Published:** 2024-08-29

**Authors:** Haomin Zhang, Quan Li, Huijuan Zhao, Bowen Wang, Jiaxing Gong, Li Gao

**Affiliations:** School of Materials Science and Engineering, Nanjing University of Posts and Telecommunications, 9 Wenyuan Road, 210023, Nanjing, China; School of Science, Nanjing University of Posts and Telecommunications, 9 Wenyuan Road, 210023, Nanjing, China

**Keywords:** computational spectroscopy, deep learning, miniature spectrometer, compressive sensing, metasurface

## Abstract

Spectroscopy is a technique that analyzes the interaction between matter and light as a function of wavelength. It is the most convenient method for obtaining qualitative and quantitative information about an unknown sample with reasonable accuracy. However, traditional spectroscopy is reliant on bulky and expensive spectrometers, while emerging applications of portable, low-cost and lightweight sensing and imaging necessitate the development of miniaturized spectrometers. In this study, we have developed a computational spectroscopy method that can provide single-shot operation, sub-nanometer spectral resolution, and direct materials characterization. This method is enabled by a metasurface integrated computational spectrometer and deep learning algorithms. The identification of critical parameters of optical cavities and chemical solutions is demonstrated through the application of the method, with an average spectral reconstruction accuracy of 0.4 nm and an actual measurement error of 0.32 nm. The mean square errors for the characterization of cavity length and solution concentration are 0.53 % and 1.21 %, respectively. Consequently, computational spectroscopy can achieve the same level of spectral accuracy as traditional spectroscopy while providing convenient, rapid material characterization in a variety of scenarios.

## 1 Introduction

In numerous scientific disciplines, spectroscopic analysis represents a fundamental and powerful tool that utilizes the absorption, emission, or scattering properties of materials in response to electromagnetic radiation (light) to reveal information about their composition and structure [1], [2]. This instrumental technique plays a pivotal role in the characterization and analysis of materials science, food science, nanotechnology, biology and chemistry, among other fields. For instance, the refractive index of thin film materials can be quantified by ellipsometry, while the characteristic absorption peaks of optical fiber materials and their quality can be readily identified by absorption spectroscopy [3]. Fourier transform infrared spectrometers are frequently employed to identify the chemical composition and solution concentration of chemical and biological samples that are commonly encountered in the food, environmental and medical industries, where characteristic absorbing and extinctive spectral features are studied [4]. Surface-enhanced Raman spectroscopy (SERS) is a highly sensitive spectroscopic method that enhances the Raman scattering of molecules and allows for the structural fingerprinting of low concentration analytes through the plasmon-mediated amplification of electric fields or chemical enhancement [5]. The extraordinary spectral resonance of plasmonics and the dielectric metasurface can be readily investigated through light transmission/reflection/scattering measurements [6]. The spectral information encodes a wealth of information not only about the material composition, but also the critical parameters of the photonic structure and cavity configuration, among other things. Consequently, it is of paramount importance to develop accurate and accessible spectroscopic techniques.

The recent rapid development and wide application of deep learning tools has enabled the direct inverse design and optimisation of photonic devices using a large amount of materials (or structures) and their corresponding spectral data [7], [8]. A deep learning approach can analyze the scattering spectra of silicon nanostructures within the diffraction-limited region and output digital information, which breaks the limits of optical information storage and already surpasses the blu-ray disc technology [9]. Recently, a deep neural network has also been employed to characterize the critical geometric and material parameters of unknown plasmonic nanostructures through simple transmission spectra [10], suggesting a simple and intelligent approach to material characterization using spectroscopic techniques.

Neverthless, high-quality spectroscopic data is typically obtained using conventional spectrometers. Conventional spectrometers typically comprise monochromators and Fourier transform spectrometers. Monochromators typically employ prisms or gratings to divide the light source into monochromatic light [11]. This process is conducted sequentially, necessitating a sufficiently long optical path to separate different wavelengths and achieve high spectral resolutions. Fourier transform spectrometers use the Michelson interferometer, which generates an interference pattern by creating a path difference between two light beams using movable mirrors [12]. The long optical length and bulky dispersive elements in these conventional spectrometers impede the adoption of low-cost, portable, fast and accurate spectroscopic analysis in everyday scenarios.

In contrast to conventional geometric optics, nanophotonic structures can extend the length of the optical path to millions of times larger than their physical dimensions. Nanophotonic structures themselves exhibit a distinctive light–matter interaction, known as the response function. Recently, with the assistance of appropriate computational algorithms based on compressive sensing theory, the spectral information can be accurately decoded and reconstructed by a multitude of miniature spectrometers [13], [14], [15]. The integration of photonic crystal structures or metasurfaces with complementary metal oxide semiconductor (CMOS) or charge-coupled device (CCD) sensors has led to the development of compact and low-cost spectral analysis tools [16], [17]. These innovations use interference patterns generated within photonic structures to encode and decode spectral information [15], [18]–[21]. Furthermore, computational spectrometers can reconstruct high-resolution spectral images from low-resolution images or sparse sampling data through deep learning, which effectively compresses spectral data, removes noise, and uses parallel computing capabilities to process large amounts of spectral data in real-time [22]–[25]. These advances facilitate the development of spectrometers with minimal signal correlation, which significantly improves the accuracy of spectral reconstruction and analysis. In addition to nanophotonic integrated image sensors, research on semiconductor nanowires has also achieved dynamic control of the photon response, making significant contributions to the miniaturization of spectroscopic instruments [26]. If the spectroscopic data can be collected by computational spectrometers, structural and material analysis can be easily decoded by deep learning algorithms, enabling a snapshot type of portable, low-cost computational characterization tool. Meng’s work has demonstrated that machine learning combined with filter arrays can be applied to material classification in theoretical simulations [27].

**Figure 1:**
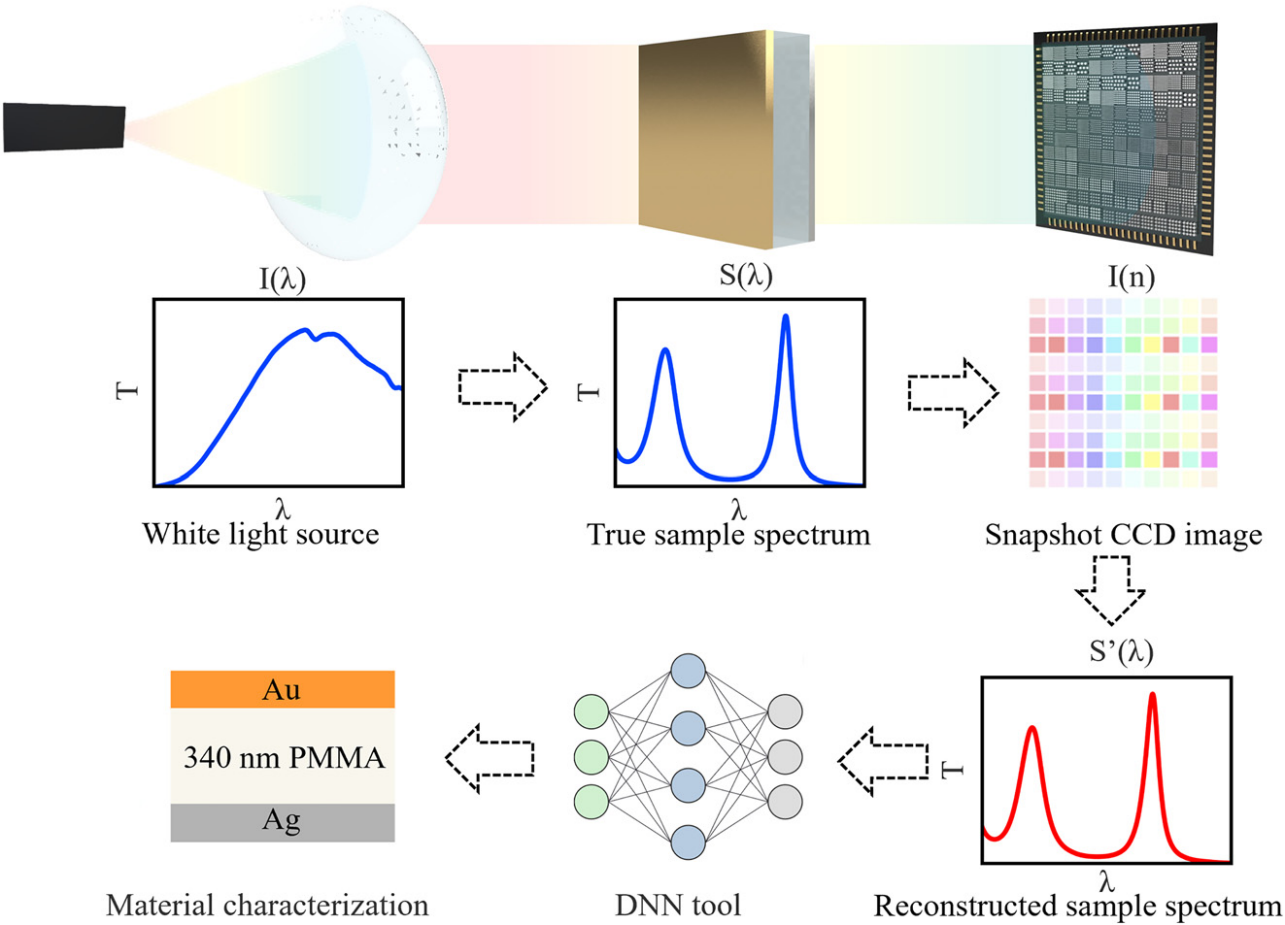
Schematics of snapshot computational spectroscopy enabled (SCS) by metasurface integrated computational spectrometer (MICS) and deep neural network (DNN). The white light source irradiates the material to be tested to form spectral information, which is then encoded by an encoder and recorded by CCD image sensor. The spectral information is then decoded by reconstruction algorithm, and finally fed into a DNN for direct characterization.

This work presents a novel snapshot computational spectroscopy (SCS) tool consisting of a metasurface integrated computational spectrometer (MICS) and a deep neural network (DNN), which is capable of rapid material analysis. The principle of operation is illustrated in Figure 1. The structural and material information of the sample of interest is contained in the transmission/reflection spectra, which are then captured by a snapshot light intensity image taken by the metasurface integrated CCD imager. The spectral information is then decoded by the reconstruction algorithm and fed into a deep neural network for direct classification and regression. All computationally reconstructed spectra achieve comparable spectral detection accuracies to those of traditional spectrometers, while the overall size of the computational spectroscopic tool is only approximately 100 mm × 50 mm × 50 mm, with a spectral sampling speed of less than 1 s. This approach provides a novel theoretical framework for the rapid and accurate prediction of material properties, while simultaneously addressing the need for portability, miniaturization, accuracy and speed of spectral acquisition and analysis.

## 2 Methods

### 2.1 The design and fabrication of metasurface filter array

**Figure 2:**
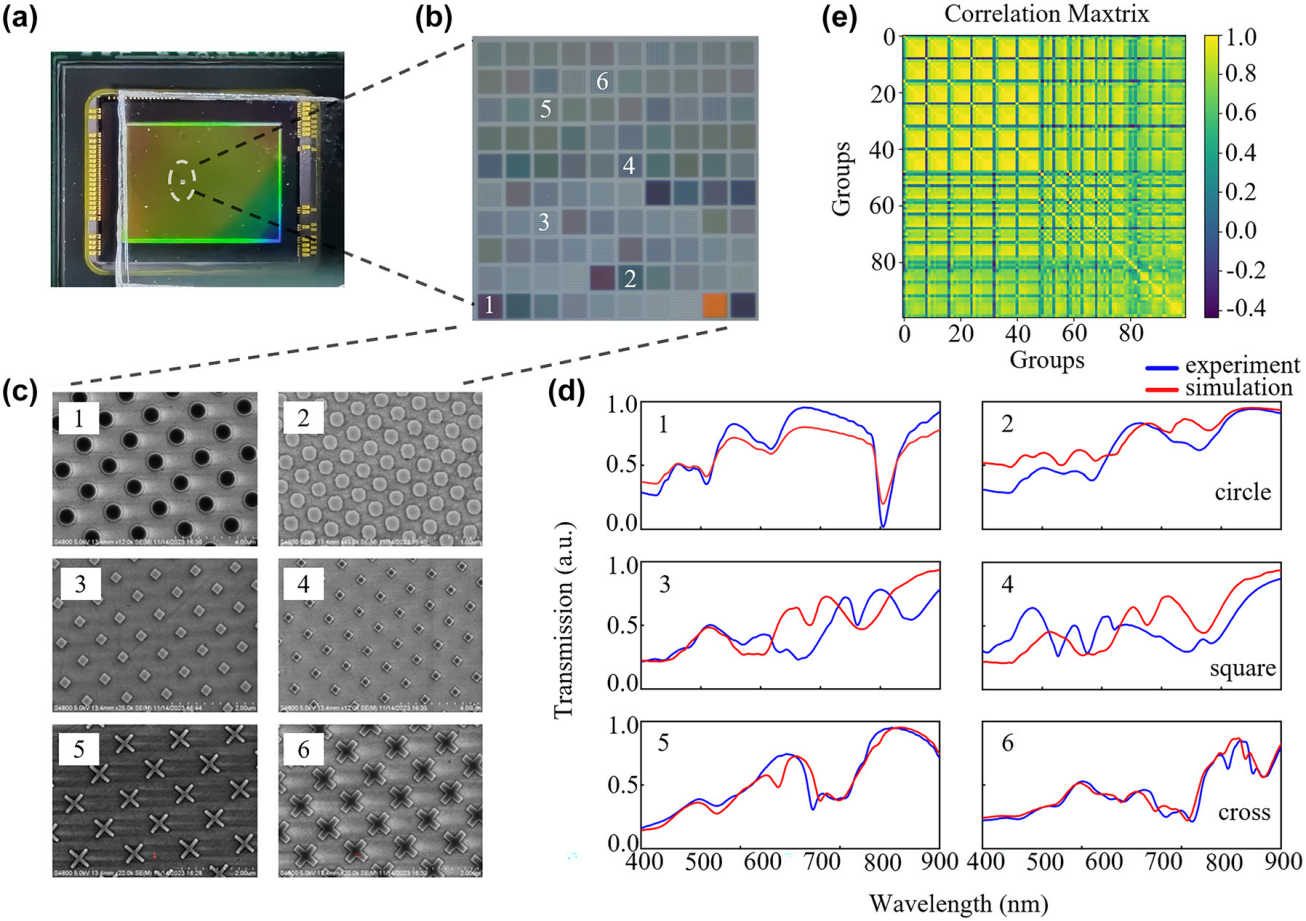
The metasurface array structures and their response functions. (a) Photograph of metasurface array mounted on a CCD image sensor. (b) An image of the 100 metasurface filter array under a microscope. (c) Scanning electron microscope of representative metasurfaces, including structures with different shapes of crosses, circles, squares, and varied diameters, periodicities, and angles between rows. (d) The simulated transmission spectra corresponding to the six structures shown in Figure c and the transmission spectra measured by MStarter ABS micro area spectrometer. (e) The correlation matrix of the overall transmission spectra of the 100 metasurface filter array.

The accuracy of spectral reconstruction is related to the size and correlation of metasurface arrays as previously discussed [28]. For better spatial resolution required in spectral imaging, a small filter array is desirable; however, this necessitates a low response function correlation for accurate spectral reconstruction. Conversely, a large filter array encodes spectra with sufficient data points, and the reconstruction accuracy is almost unaffected by the array correlation coefficient if the array size is greater than 7 × 7 structures from previous investigations [28], [29], [30]. In our work, in order to achieve a high level of spectral reconstruction accuracy, we selected a 10 × 10 large array structure as an encoder from the filter design database based on our previous inverse design approach. Afterwards, a thin layer of UV glue is spin coated on the surface of the CCD sensor to bond the processed metasurface array chip (with sapphire substrate) to the sensor surface after ultraviolet radiation. The metasurface array chip can thus be integrated onto the CCD sensor as illustrated in Figure 2(a) and (b).

In order to optimize the array design, the Lumerical FDTD Solutions software was employed to obtain the filter design database, which contains five hundred metasurfaces with different transmittances (response functions) resulting from different shapes, sizes, orientations and periods of the meta-atoms. This is illustrated in Figure 2(c). This metasurface database is relatively small as we need to consider the nanofacrication feasibility and effective response in the visible domain. In order to select the optimized array structure with the least correlation in this relatively small database, the parameterized design of the metasurface was fed into a deep neural network (DNN) along with the corresponding transmission spectra, as show in Figure S1(a). The loss function was defined to minimize the discrepency between the model output and target output, including a regularization term to assess the correlation of different structural groups. The transmission spectra of all designs are computed by forward propagation through the network, and an algorithm is used to adjust the parameters of the metasurface structures to minimize the loss function. Through this method, we are able to select 100 filter designs with the lowest correlation from the validation results and the final array exhibits an average correlation value of 0.681 which is sufficient for accurate spectrum reconstruction [28]. In addition, the actual correlation value deviates from the design a bit due to unavoidable nanofabrication error induced spectral response discrepancy, our simulation correlation is 0.57.

Following the selection the structural parameters, the sapphire substrate was coated with a 100 nm thick silicon film through plasma-enhanced chemical vapor deposition (PECVD), which was identical to the simulated conditions. Each fabricated metasurface filter in the array is approximately 15 μm in size, with a 2 μm spacing between each other, resulting in a total array size of 170 μm × 170 μm. The metasurface patterns are transferred to the silicon film by electron beam lithography (EBL) followed by reactive ion etching (RIE) to obtain the ultimate metasurface filter array chip. Subsequently, the transmission spectra of each metasurface in the fabricated encoder chip was measured by using MStarter ABS with a spot size of 5 μm and a wavelength range from 400 nm to 900 nm. Figure 2(d) illustrates the simulated and experimental response functions for six representative metasurface structures. There is unavoidable spectral response discrepancy between the simulation and fabrication due to unavoidable nanofabrication errors. All the 100 transmissive response functions are converted into a response matrix equation *T*_*i*_(*λ*), and the actual measured array correlation is calculated and presented in Figure 2(e).

### 2.2 The calibration of computational spectrometer and spectral reconstruction

In the construction of a miniaturized computational spectrometer, a white LED point light source is employed for the purpose of system miniaturization. In order to convert the scattered light from the point source into uniform, parallel light, a thin convex lens is installed behind the light source. The thin convex lens is capable of shaping the light into parallel beams, which are necessary for subsequent CCD signal collection. Given that there is always a photon loss during the photosensing process, it is of the utmost importance to calibrate CCD light intensity sensitivity, denoted by *η*(*λ*) prior to reconstruction. The calibration ultilizes a monochromator with its optical path illustrated in Figure S2(a). The operational mode may be either reflection or transmission, as illustrated in Figure S2(b) and (c). The CCD industrial camera model we use is MV-CU120-10 GM, with a single pixel size of 1.85 μm × 1.85 μm. The response curve as shown in Figure S3. The composition or structure of the sample may either absorb or scatter light, resulting in a unique spectral fingerprint. Subsequently, the spectral information is collected by the CCD image and converted into an electrical signal for subsequent decoding processing, following its entry into the metasurface array encoder. For calibration purposes, the monochromator was employed to sequentially scan the wavelength range from 400 nm to 900 nm with a 0.4 nm bandwidth. At each wavelength, the light intensity signals were collected using a CCD and a photometer. The incorporation of the light intensity sensitivity *η*(*λ*) of the CCD image sensor, calculated and incorporated into the reconstructed spectrum, can result in a more realistic and accurate signals, thereby improving the accuracy of spectral reconstruction. Finally, the incident spectrum *I*(*λ*) can be reconstructed using the collected CCD image sensor’s electrical signals *S*_*i*_, the response matrix equation *T*_*i*_(*λ*), and the spectral sensitivity *η*(*λ*) through the spectral reconstruction formula (1), as previously described in numerous works [14], [16], [17], [31]. The detailed principle can be obtained in Figure S4.

(1) $$S_{i}=T_{i}\left(\lambda \right)\;*\;\eta \left(\lambda \right)\;*\;I\left(\lambda \right)$$

### 2.3 The training of deep neural network for spectral characterization

Furthermore, in order to perform computational characterization with high accuracy and efficiency, a DNN network was constructed. The DNN comprises of a 1 × 1,251 input layer for the reconstructed spectra from 400 to 900 nm, 3 × 2,000 fully connected hidden layers, a 1 × *n* regression network output layer, and a 1 × *m* classification network output layer (where *n* is the number of labels required for the regression network output, and m is the number of labels for the classification network output). The activation function of the regression network is the rectified linear unit (ReLU), while that of the classification network is the Softmax function. The DNN neural network architecture is shown in Figure S1(b). As demonstrated in our previous research, experimental and simulated spectra exhibit common resonance features. An accurate experimental characterization of the material property was demonstrated using DNN training on mixed experimental data and simulated data [10]. Learning from this, we have used only simulated data for DNN training and the test dataset consists only of experimental data. In this way, the burden of collecting sufficient experimental data as a training dataset is greatly reduced.

## 3 Results

### 3.1 The spectral reconstruction accuracy analysis

**Figure 3:**
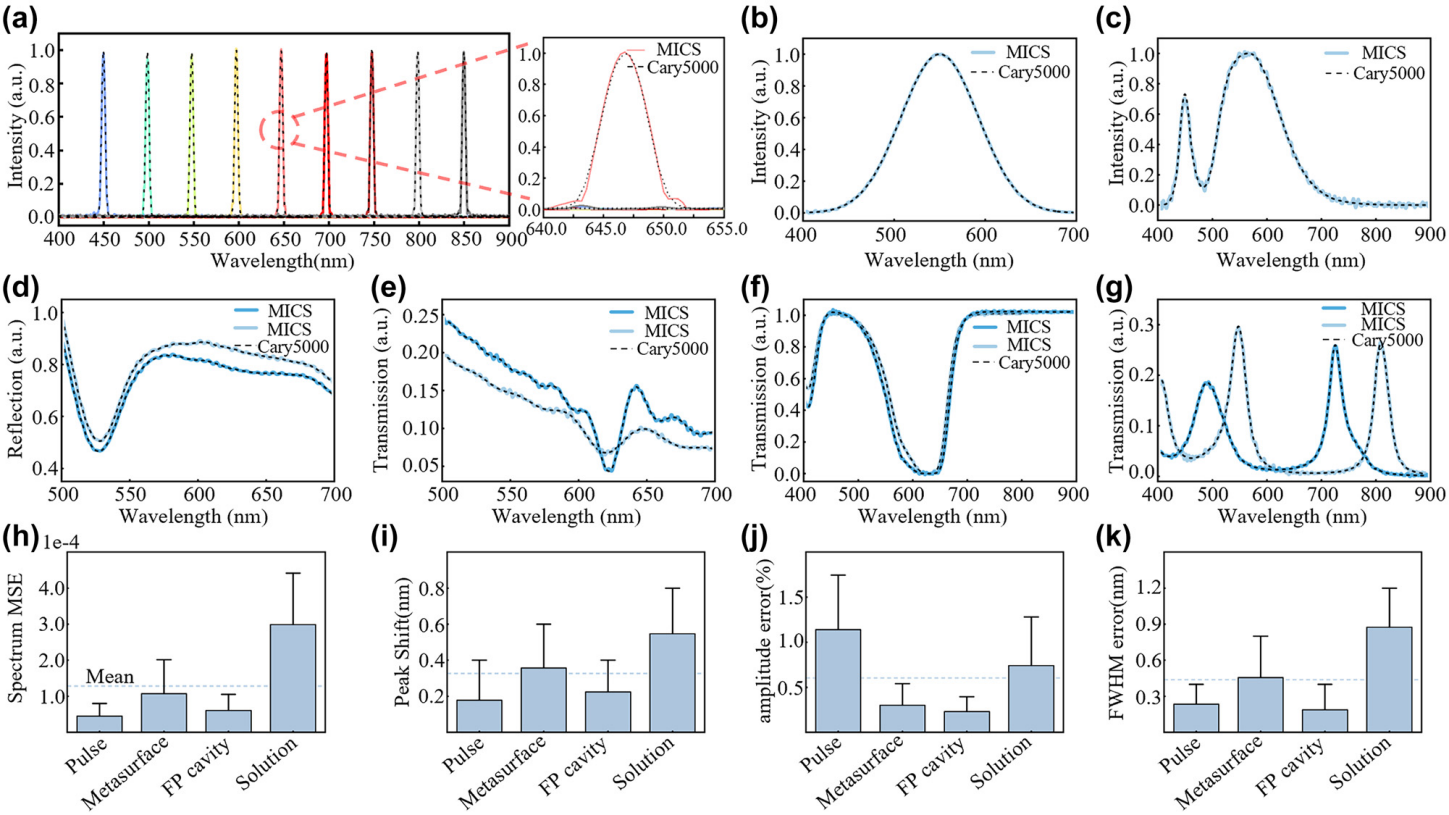
Spectral reconstruction accuracy analysis. (a) The left image shows the spectra of pulse light with a full width at half maximum (FWHM) of 4 nm and intervals of 50 nm in the 400 nm–900 nm range, detected by both traditional spectrometers and miniature computational spectrometers. The dashed lines represent the results from Agilent Cary5000, while the solid lines are the results from MICS. The right image shows the enlarged spectral detection results for the pulse spectrum centered at 650 nm. (b) And (c) measured and reconstructed spectra of two different light sources. (d) Transmission spectra of a metasurface before and after spin-coating of an optical adhesive NOA 63, detected by both types of spectrometers. (e) The reflection spectra of two optical metasurfaces with different periods and diameters. (f) The transmission spectra of the methyl blue solution concentration with a difference of 0.01 g/L. (g) The transmission spectra of metal-dielectric-metal multilayer film with different dielectric layer thicknesses. (h)–(k) Mean squared error of spectrum, average peak shift error, average amplitude error and FWHM error in (a), (d)–(g).

In order to verify the precision and accuracy of our miniaturized computational spectrometer, we collected spectral information from each sample using both high-precision commercial UV–Vis–NIR spectrometer (Agilent Cary5000) and our MICS approach. First, we validated the reconstruction accuracy of simple spectra from narrow pulse spectra in the 400 nm–900 nm wavelength range. The narrow pulse spectra were obtained over a wavelength range of 450 nm–850 nm with a 50 nm pulse interval and a spectral full width at half maximum (FWHM) of 4 nm, as shown in Figure 3(a). The dashed lines represent the transmission spectra measured by the Cary5000, while the solid lines represent the transmission spectra reconstructed by MICS. An enlarged spectral peak comparison of a 650 nm pulse signal is shown on the right. The reconstructed spectra of incandescent and LED light sources have high reconstruction accuracy, as shown in Figure 3(b) and (c). We also reconstructed a narrow spectrum with a peak shift difference of only 0.4 nm, demonstrating that our spectral reconstruction accuracy reached 0.4 nm, as shown in Figure S5.

In order to validate the spectral reconstruction accuracy and resolution for actual nanophotonic structures and chemical solutions, a number of cases have been selected for test and comparison. The first group comprises two metasurfaces comprising circular nanoposts with a period of 1,300 nm and a diameter of 700 nm. One of these metasurfaces has been spin coated with a thin layer of NOA 63 with a refractive index value of 1.56, situated above the metasurface (Figure S6(a)). The transmission spectra of each sample were measured and are shown in Figure 3(d). The dashed line represents the measurement by the Cary5000, while the solid line represents the measurement by MICS. The second group comprises circular nanopost metasurfaces with the following parameters: Period = 400 nm, Diameter = 300 nm, and *P* = 300 nm, *D* = 200 nm (Figure S6(b)). Their transmission spectra are shown in Figure 3(e). The third group is composed of methyl blue solutions with a concentration difference of 0.01 g/L (Figure S6(c)), and its transmission spectrum is shown in Figure 3(f). The fourth group is composed of a simple metal-dielectric-metal multilayer film configuration, where the top metal layer is Ag, the bottom layer is Au, and the insulator layer is methyl methacrylate (MMA). The dielectric thickness of the insulator layer is 280 nm and 340 nm, respectively (Figure S6(d)). Their transmission spectra are shown in Figure 3(g). A direct comparison of the reconstructed and measured spectra reveals that the computational spectrometer is capable of accurately reproducing the spectral characteristics of the measured data.

A quantitative analysis was conducted on four aspects of the above spectra, including the mean square error (MSE), peak shift error, amplitude error and full width at half maximum (FWHM) error in reconstructed spectra. The results presented in Figure 3(h) – (k) demonstrate that the average MSE for all spectral reconstructions was 1.28 × 10^−4^, the average peak shift error for all reconstructed spectra was 0.32 nm, and the average peak amplitude error was 0.6 %, the FWHM error is 0.437 nm. The analysis revealed that the reconstruction offset error of a miniaturised computational spectrometer was 0.32 nm, which was smaller than its spectral resolution of 0.4 nm. Consequently, the reconstruction accuracy of MICS is 0.4 nm, with a reconstruction accuracy of 99.4 %, closely matching the spectra detected by traditional spectrometers. Within the current study range of 400 nm–900 nm, the MICS can be employed as a substitute for traditional spectrometers for spectral analysis.

### 3.2 Material and structural computational characterization

The application of the SCS technique to the characterisation of a simple optical cavity is demonstrated, based on the highly accurate spectral information sensed by the MICS technique. The Fabry–Pérot (FP) cavity is a common optical cavity employed in a variety of laser, spectrometer, and optical instrument applications. An FP cavity is composed of two parallel mirrors and an adjustable cavity length. This phenomenon of wavelength-dependent interference can either enhance or diminish the transmission spectrum, resulting in the formation of peaks. The characterisation of the FP cavity length typically relies on sophisticated tools such as scanning or tunneling electron microscopes for cross-sectional examination. The precise relationship between the cavity length and the peak wavelength can be expressed as 2nd = *mλ*, where *n* is the refractive index within the cavity, *d* is the cavity thickness, *m* is an integer, and *λ* is the wavelength of the transmitted light. If the total path length is an integer multiple of the wavelength of the light, the reflected light waves will constructively interfere, forming interference peaks.

**Figure 4:**
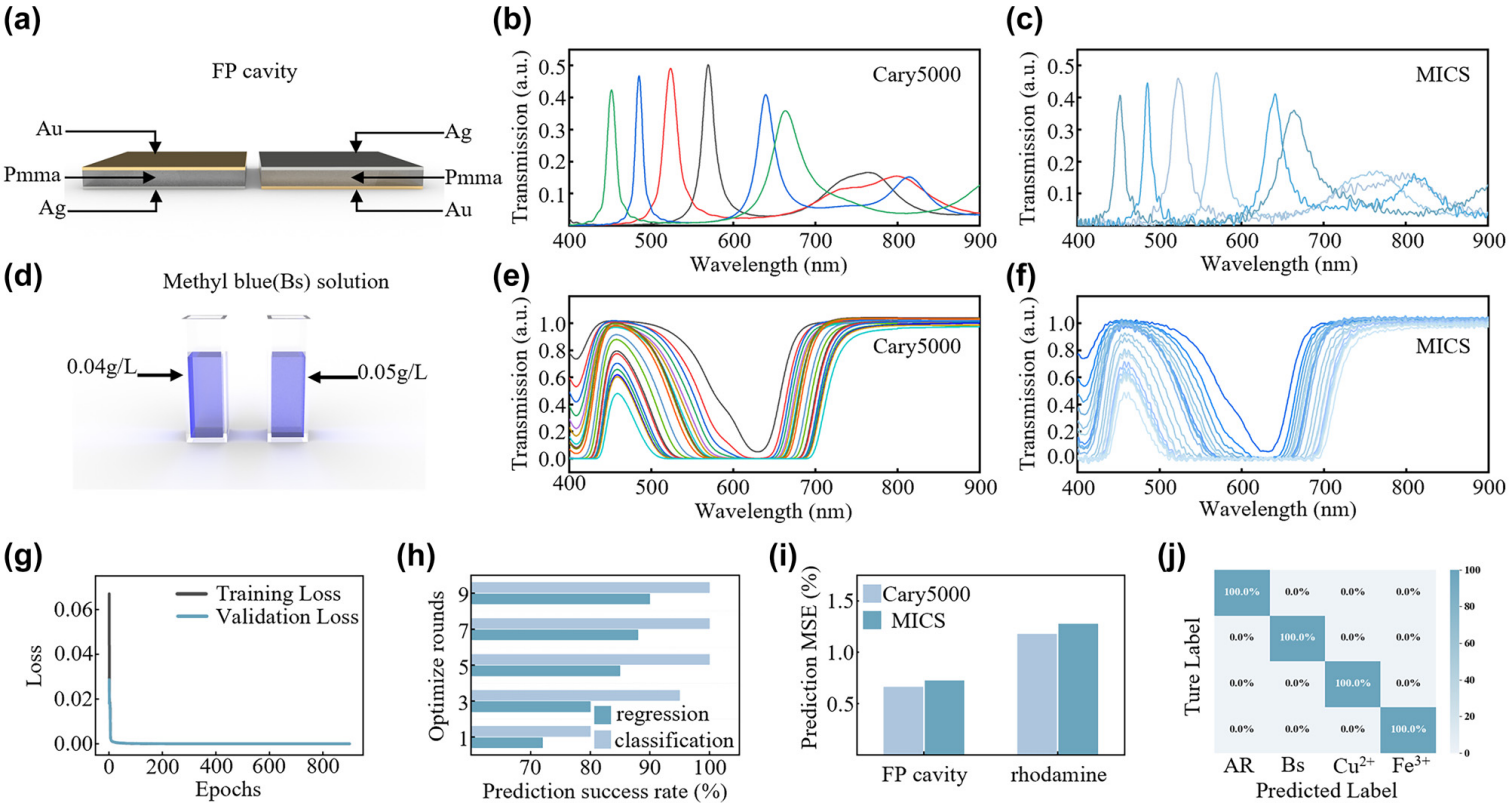
The application of snapshot computational spectroscopy. (a) Schematic diagram of the Fabry–Pérot (FP) cavity. (b) Transmission spectra of FP cavities with different dielectric cavity lengths measured by Carry5000. (c) Transmission spectra of FP cavities with different dielectric cavity lengths measured by MICS. (d) Schematic diagram of the solution samples with different concentrations. (e) Transmission spectra of methyl blue solutions with different concentrations measured by Carry5000. (f) Transmission spectra of methyl blue solutions with different concentrations measured by MICS. (g) Loss curve after the DNN network training is complete. (h) Changes in the success rate of prediction for the regression and classification networks during the hyperparameter optimization process. (i) Statistics of the mean squared error between the predictions from data obtained from Cary5000 and MICS. (j) Confusion matrix for predicting solute types using classification networks.

In accordance with the constructive interference condition, as the cavity length of the FP cavity increases, the wavelength supporting constructive interference shifts to longer wavelengths, as illustrated in Figure 4(b). Consequently, the identification of the peak positions of the transmission spectra allows for the characterisation of the cavity length. As deep neural networks (DNNs) can map the physical relationship between structure and spectra with high accuracy, we have incorporated a DNN to analyse the reconstructed spectra, with the aim of predicting the material type, cavity length and cavity configuration, among other things, in an intelligent manner. The initial training set comprised three-layer FP cavities simulated using Lumerical FDTD Solutions, with varying thicknesses and materials. The training set included two metals (Au, Ag) and three dielectrics (PMMA, MMA, Al_2_O_3_) with dielectric layer thicknesses ranging from 300 nm to 900 nm, at 10 nm intervals, totalling 26,000 datasets. The degree of fit between the simulated and experimental spectra was found to be between 91.2 % and 98.0 % after data normalization and preprocessing, as illustrated in Figure S7–S9. The entire set of simulated data was employed as the training set for the DNN network, with the trained DNN subsequently used for the prediction of experimental spectra.

Subsequently, 100 sets of FP cavities with different metals, dielectrics, and thicknesses were measured using MICS and Cary 5000, which served as the test set for the pre-trained DNN network to analyze its prediction success rate. The DNN network has two outputs: one is the classification network output, which is designed to predict the type of metal and dielectric material present in the FP cavity, and the other is the regression network output, which is intended to predict the thickness of the FP cavity dielectric layer. The prediction performance for different dielectric layer thicknesses is illustrated in Figure S10(g) and (h). The dielectric layer of aluminum oxide exhibited the highest MSE, with a prediction error of 0.76 nm by Cary 5000 and 0.81 nm for MICS. The lowest mean square error (MSE) was observed for MMA, with a prediction error of 0.23 nm for Cary 5000 and 0.25 nm for MICS, respectively. Both types of spectrometers achieved a 100 % success rate in predicting the type of materials, as demonstrated in Figure S10(a)–(f), which present the confusion matrices for the predictions made by the two spectroscopic data sets.

Another typical application example is the spectroscopic analysis of chemical solutions. When light passes through a solution, a portion of the light is absorbed by the solute present within the solution. The quantity of light absorbed is contingent upon the nature and concentration of the solute, and this relationship is elucidated by Beer’s Law. The law states that the absorption of light at a specific wavelength is directly proportional to the concentration of the solute, with the formula *A* = *ϵ* × *c* × *l*, where *A* is the absorbance, *ϵ* is the molar absorptivity, *c* is the molar concentration of the solute, and *l* is the path length of the light through the solution. Transmission spectra demonstrate the intensity of light that has traversed the solution, reflecting the quantity of light that has not been absorbed. Figure 4(e) illustrates the manner in which the transmission spectrum of a methylene blue solution varies with concentration. Given that different solutions exhibit varying absorption coefficients, transmission spectra can be employed to ascertain the nature and concentration of solutions. In the absence of knowledge regarding the concentration of the solution, it is necessary to characterise the solution’s properties using a range of common methods, including spectrophotometry, titration, conductivity determination and nuclear magnetic resonance spectroscopy, among others.

In order to illustrate the SCS technique in chemical solution characterisation, four types of solutions were selected, each containing a different chemical: methylene blue (Bs), rhodamine (AR), Cu^2+^ ions and Fe^3+^ ions. The concentrations of Bs and AR ranged from 0.01 g/L to 1 g/L, while those of Cu^2+^ and Fe^3+^ ranged from 1 g/L to 100 g/L, resulting in a total of 80 datasets. All the measured experimental spectra were used for training purposes. In order to expand the dataset size, random errors were added for data augmentation, resulting in a total of 720 datasets. Of these, 80 % were selected for training, while the remaining 20 % were used for testing. As the selection of hyperparameters can directly impact the performance and efficiency of models in deep learning and cannot be automatically updated during training, we also incorporated hyperparameter optimisation. The hyperparameters that were optimized included the number of neurons, the activation function, the number of epochs, the batch size, and the number of optimization rounds, which were set at 10. Hyperparameter optimization can markedly enhance model performance, prevent overfitting, accelerate training, and accommodate diverse data characteristics.

Figure 4(i) illustrates the efficacy of the classification and regression networks during hyperparameter optimization. A regression network for the FP cavity is considered successful if the predicted thickness error is less than 1 nm from the actual thickness error. For solutions, a success is defined when the concentration error is less than 2 %. The DNN network loss curve is depicted in Figure 4(h). Upon inputting the test dataset of solutions into the trained DNN network, the prediction results for varying solution concentrations are presented in Figure S11(c) and (d), with an overall concentration mean square error of 1.8 %. The results of solution type predictions by both the Cary 5000 and MICS are shown in Figure S11(a) and (b), respectively, with 100 % accuracy. The overall mean squared error (MSE) for the FP cavities and solutions is displayed in Figure 4(j), which demonstrates that the chemical solution characterisation by both spectrometers exhibits negligible differences in the predicted errors.

## 4 Discussion

The operational scope of traditional spectrometers is contingent upon the spectral range of gratings and photodetectors, whereas computational spectrometers are also constrained by the dynamic range of encoders and image sensors. The operational range of the computational spectrometer described in this article extends from 400 nm to 900 nm, corresponding to the photosensitive range of the CCD image sensor and the design of the metasurface array encoder. However, this does not preclude the potential applications of this MICS technology in the ultraviolet and infrared bands. Furthermore, in order to expand the application of the SCS technique in reality, it is necessary to collect sufficient spectral data for various materials in order to form pretrained DNN tools. This necessitates the acquisition of a considerable quantity of experimental spectral data and the subsequent training of DNNs.

## 5 Conclusion

The combination of a MICS hardware and a DNN software tool can form a snapshot computational spectroscopic technique, which can rapidly and accurately identify a sample’s critical properties such as the DNN network and chemical solution concentration. This represents an innovative alternative to traditional spectrometers and provides a new theoretical foundation for the rapid and precise characterisation of materials. In the future, by optimising the design and fabrication of encoders and photodetectors, the sampling range of miniature computational spectrometers can be broadened to the ultraviolet and infrared regions, with the potential for further miniaturisation. The integration of a metasurface with two-dimensional photodetectors enables the realization of an ultraminiature configuration with micrometer dimensions. The technology is compatible with the CMOS technology, which facilitates the integration of MICS within a smartphone. This integration enables the development of truly portable SCS applications for spectral detection and material characterization in a range of biomedical, food and environmental test scenarios.

## Supporting Information

Supporting information includes spectral reconstruction algorithm, spectral detection of different substances, feasibility analysis of FP cavity prediction and analysis of prediction results of the DNN networks.

## Supplementary Material

Supplementary Material Details
