# Supplementary material for: Snapshot computational spectroscopy enabled by deep learning
Source: Nanophotonics. 2024 Aug 29;13(22):4159–68. doi: 10.1515/nanoph-2024-0328 (PMC11501049; doi:10.1515/nanoph-2024-0328)
Supplement: Supplementary file 1 — Supplementary Material Details [file j_nanoph-2024-0328_suppl_001.docx]

**Supporting Information**

**Snapshot computational spectroscopy enabled by deep learning**

**Haomin Zhang, Quan Li, Huijuan Zhao^*^, Bowen Wang, Jiaxing Gong, Li Gao^*^**

**1 Schematic diagram of neural network structure**


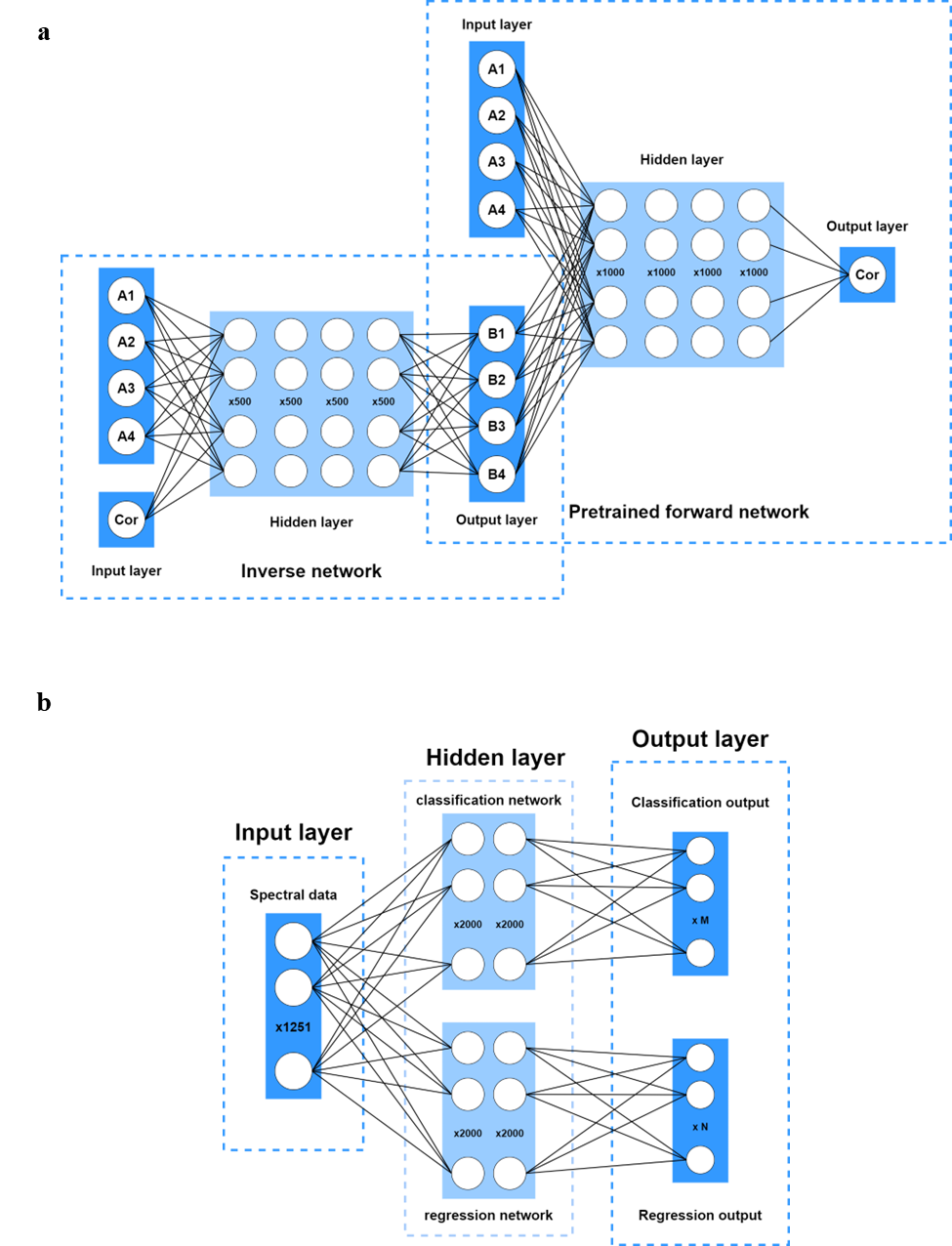


**Figure S1.** Schematic diagram of the neural network structure. (a) Inverse design network architecture diagram, the tandem network consists of the trained forward network on the right connected to the inverse network. The forward network has 8 input parameters, namely the diameter, gap, shape and angle of the first and second structures, and outputs a correlation coefficient value. The inverse network has 5 input parameters, which are the four parameters of the basic structure and a correlation value, and the output is the four parameters of the new structure. The parameters of the base structure and the new structure are input into the forward network. (b) Schematic diagram of the DNN neural network structure for predicting material information.

**2** **Optical path diagram**

**
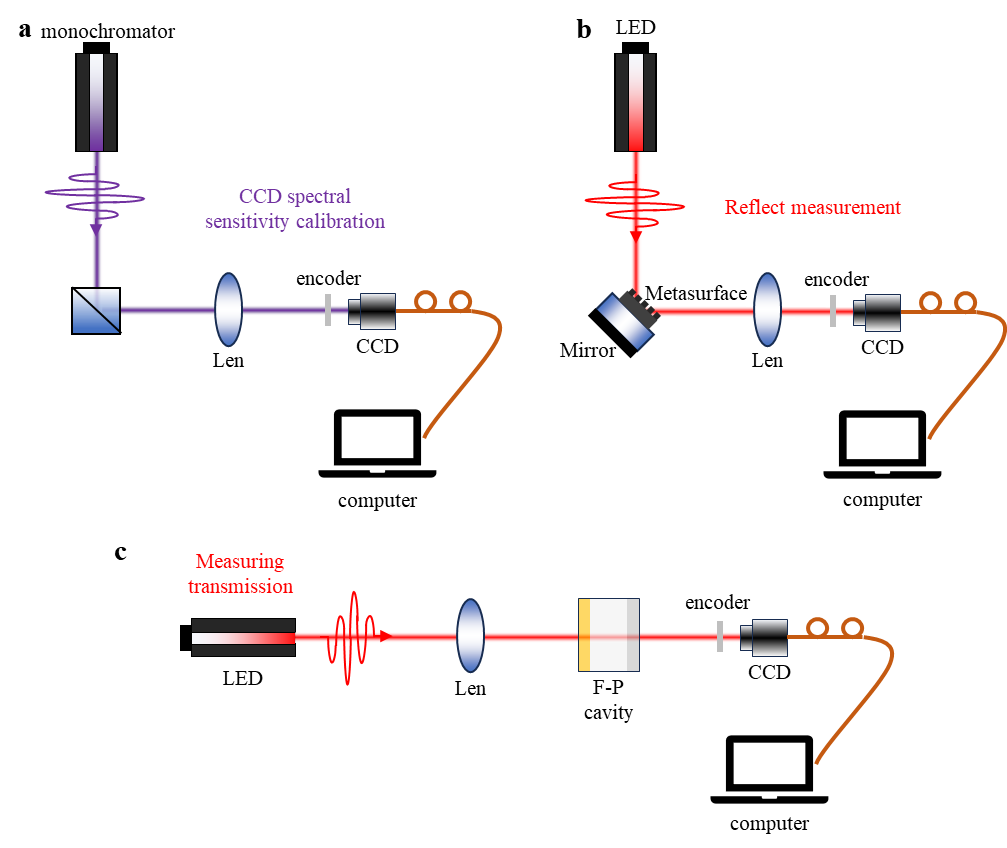
**

**Figure S2.** Experimental optical path diagram. (a) Calibration of CCD spectral sensitivity using a monochromator. (b) The optical path for measuring the reflection spectrum of substances. (c) The optical path for measuring the transmission spectrum of substances.

**3 Alignment scheme between metasurface array and CCD pixels**

After fixing the light path, use parallel white light sources to illuminate the integrated chip. After obtaining the image information, it is converted into a grayscale value matrix, taking into account the possible refraction effects between different metasurfaces and the errors caused by the metasurfaces at the edges not covering the entire pixel. In the end, we chose the center of each metasurface as a 5 × 5 pixel unit, which is 9.25 μm × 9.25 μm, as the fixed sampling area. At this point, the alignment problem between the array and CCD pixel units has been completed.

*
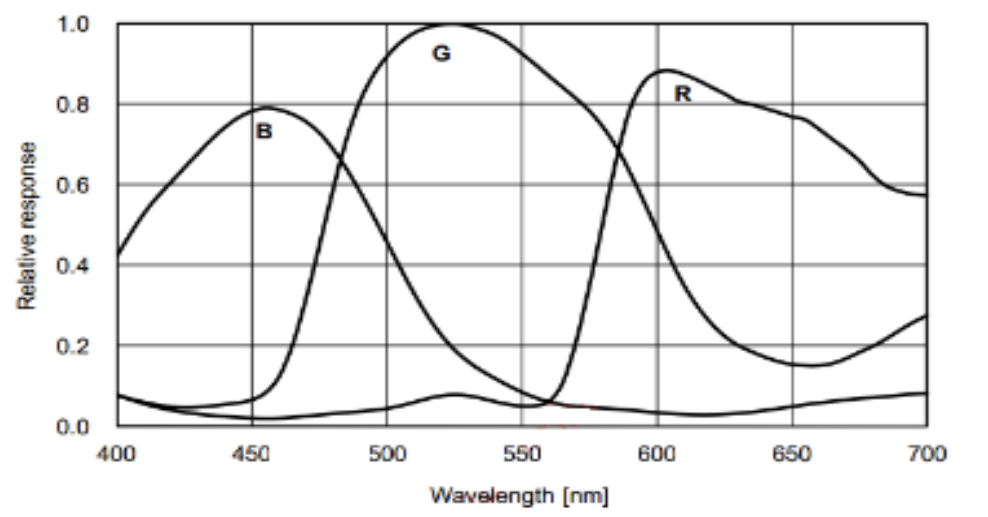
*

**Figure S3**. response curve of the MV-CU120-10GM.

**4** **Spectral reconstruction algorithms**

The integration formula for spectral reconstruction is:

$S_{i}=\int T_{i}(\lambda)\eta(\lambda)I(\lambda)\text{d}\lambda$ (S1)

$S_{i}(i=1,2\cdots,m)$ is the electrical signal collected by the i-th metasurface through a CCD image sensor, $T_{i}(\lambda)(i=1,2\cdots,m)$ is the transmission spectrum corresponding to the i-th metasurface. $\eta(\lambda)$ is the calculated spectral sensitivity of CCD, $I(\lambda)$ is the spectrum of the sample to be tested. Since the response matrix $T(\lambda)$calibrated in advance is not a continuous function, the solved $I(\lambda)$ contains only limited spectral information. Therefore, a series of Gaussian basis combinations is used to represent:. where $\alpha_{j}(j=1,2\cdots,n)$ is the constant coefficient, is a Gaussian function with a peak. Then, Equation (S1) can be written as:

(S2)

Converting the integral equation to linear algebra:$S=K\alpha$ yields the formula:

$\left[\begin{matrix} S_{1} \\ S_{2} \\ \vdots\\ S_{m} \end{matrix}\right]=\left[\begin{matrix} K_{1}(\lambda_{1}) & K_{1}(\lambda_{2}) & \cdots& K_{1}(\lambda_{m}) \\ K_{2}(\lambda_{1}) & K_{2}(\lambda_{2}) & \cdots& K_{1}(\lambda_{n}) \\ \vdots& \vdots& \ddots& \vdots\\ K_{n}(\lambda_{1}) & K_{n}(\lambda_{2}) & \cdots& K_{n}(\lambda_{m}) \end{matrix}\right]$ (S3)

In order to achieve better resolution, the number of detectors or filters in our broadband filtering system is smaller than the number of sampled wavelengths (m<<n), and a solution range limitation is added to make the calculated spectrum closer to the actual spectrum


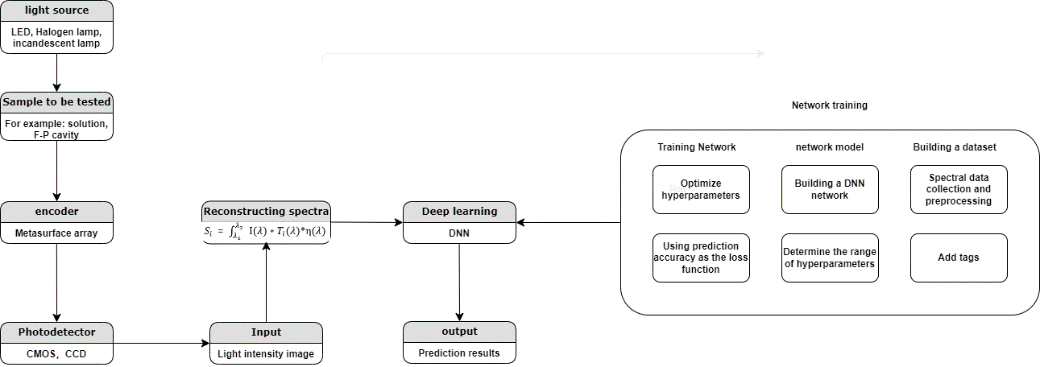


**Figure S4.** Spectral Reconstruction and DNN Prediction Network Flowchart.

**5 Proof of spectral resolution**

To demonstrate the spectral resolution of MICS at 0.4 nm, we supplemented a set of spectral reconstructions of pulse spectra with peak shifts of only 0.4 nm, as shown in Figure S5.


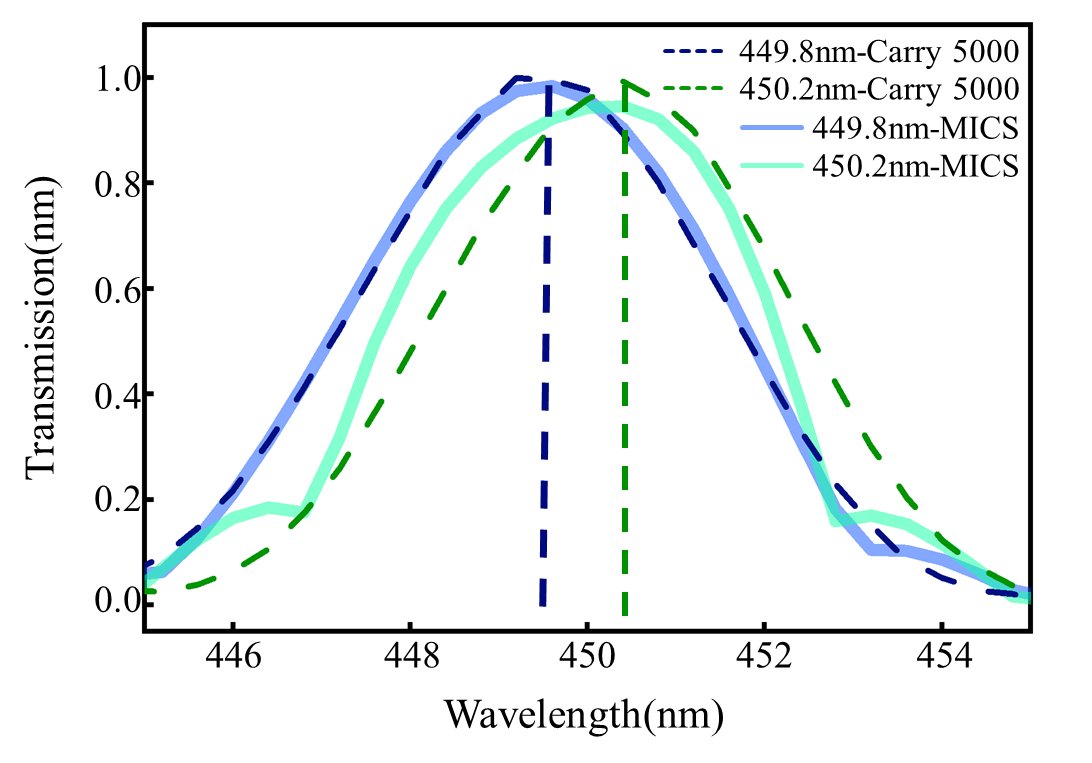


**Figure S5.** Narrow pulse spectrum with peak difference of 0.4nm. The peak positions of the two narrow pulses are 449.6 nm and 450.0 nm, respectively.

**6 Schematic diagram of different substances**

**
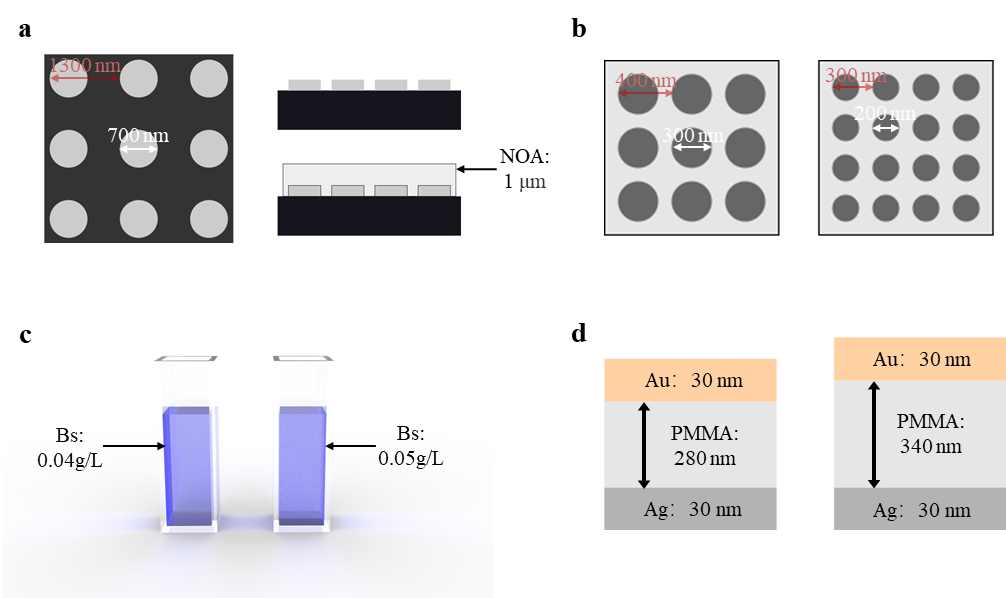
**

**Figure S6.** Schematic diagram of four groups of structures. (a) Schematic diagram of the structure of the first group of metasurfaces. (b) Schematic diagram of the structure of the second group of metasurfaces with different parameters. (c) Schematic diagram of the third group with different solution concentrations. (d) Schematic diagram of FP cavities with different cavity lengths in the fourth group.

**7** **Feasibility analysis of FP cavity prediction**

To verify that the F-P cavity can be trained using simulation data instead of experimental data, we first selected ten sets of F-P cavities with the same parameters, simulated their transmission spectra using Luminary FDTD Solutions, and measured their transmission spectra using an Agilent spectrometer as shown in the Figure S7a,b. From the figure, it can be seen that the main difference between the two transmission spectra is the spectral peak, and the peak positions and bandwidths of the spectra are consistent. In order to better align the simulation results with the actual conditions, we performed normalization. As a result, the mean squared errors of the two groups decreased from 0.148 and 0.160 to 0.08 and 0.02, respectively, as shown in Figure S7c,d.

To verify the feasibility of our prediction of substances, it is necessary to first ensure that the spectral differences of different parameters are greater than the resolution of a micro computational spectrometer. We use the method of controlling variables to change only the top metal material, as shown in Figure S8a for spectral differences, and only the bottom metal material, as shown in Figure S8b for spectral differences. The transmission spectra of the dielectric layer with thicknesses of 400 nm and 401 nm were measured separately. The spectral peak shift caused by the difference in 1 nm thickness is 1.646 nm, as shown in Figure S8c. The transmission spectra of different concentrations of four solutions were measured as shown in Figure S9. The spectral differences between different solutions are significant, and the same solution with different concentrations also maintains spectral differences of over 1 nm.

In summary, it can be concluded that for the FP cavity and solution, the spectral differences corresponding to different parameters are much greater than the minimum resolution of 0.32 nm of the micro computational spectrometer.


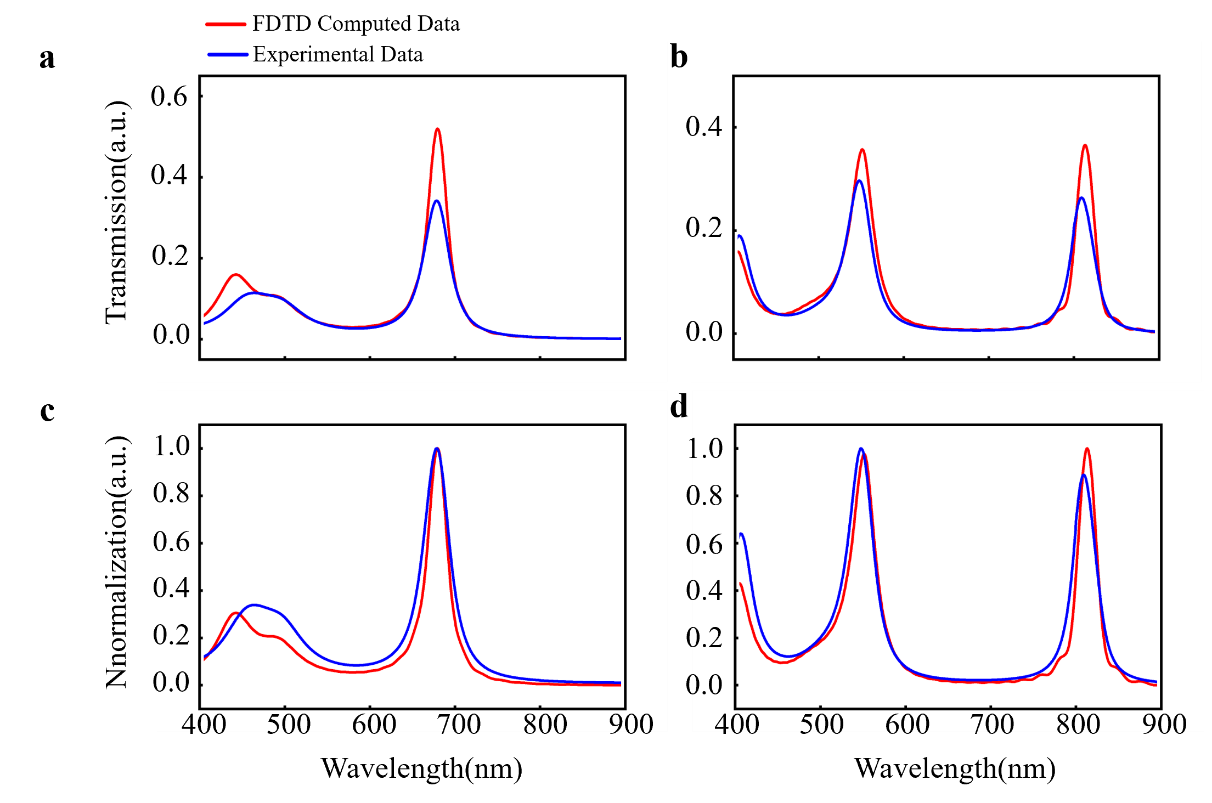


**Figure S7.** Spectral comparison between simulation and experiment before and after normalization. (a) , (b) Before normalization, two sets of simulation and experimental F-P cavity spectra were obtained. (c) , (d) Normalized F-P cavity spectra from two sets of simulations and experiments.


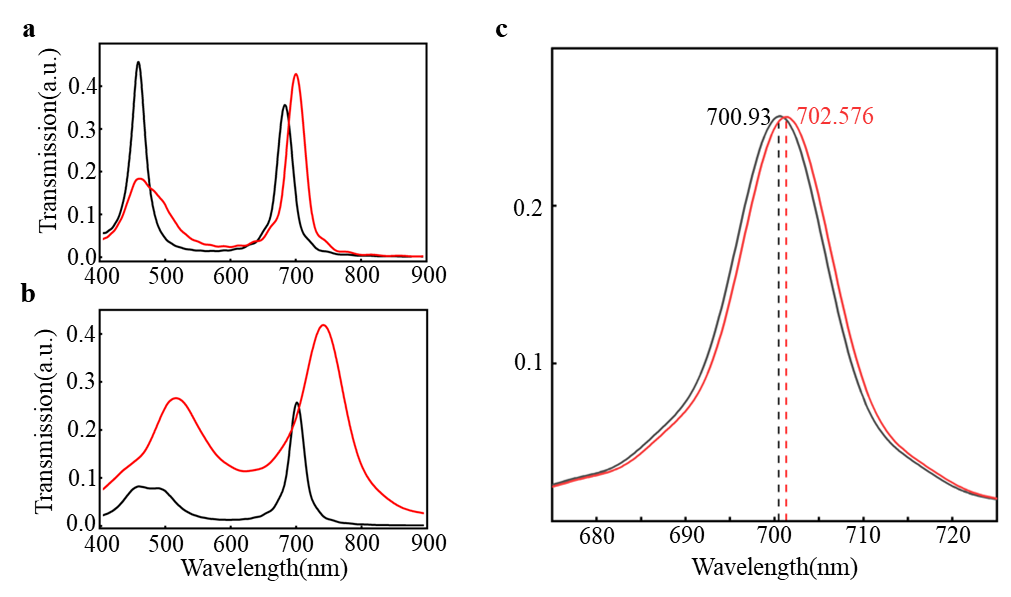


**Figure S8.** Spectra of F-P cavity with different parameters. (a) Transmission spectra corresponding to different metals on the top layer. (b) Transmission spectra corresponding to different metals at the bottom layer. (c) Transmission spectra corresponding to different thicknesses of the dielectric layer.


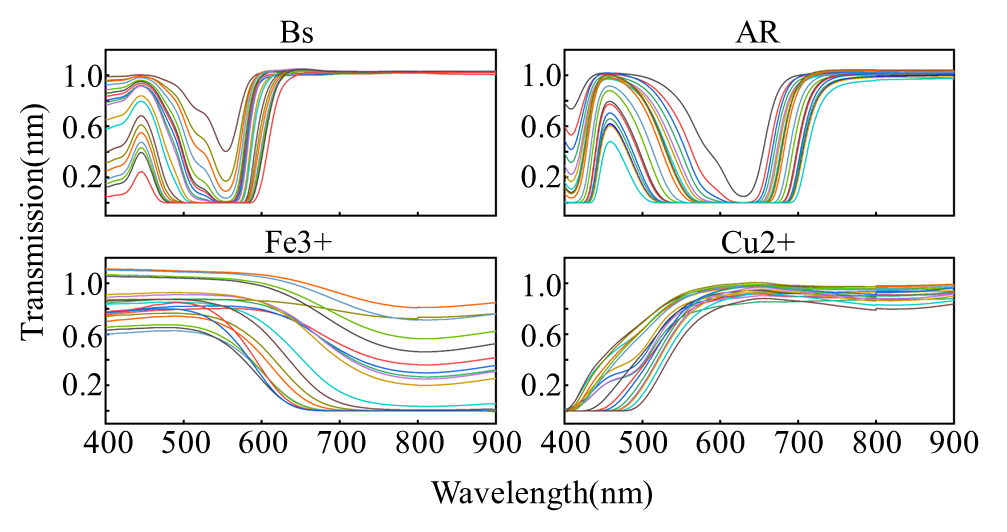


**Figure S9.** Spectral changes corresponding to four solute solutions at different concentrations

**8 Analysis of prediction results of DNN networks**


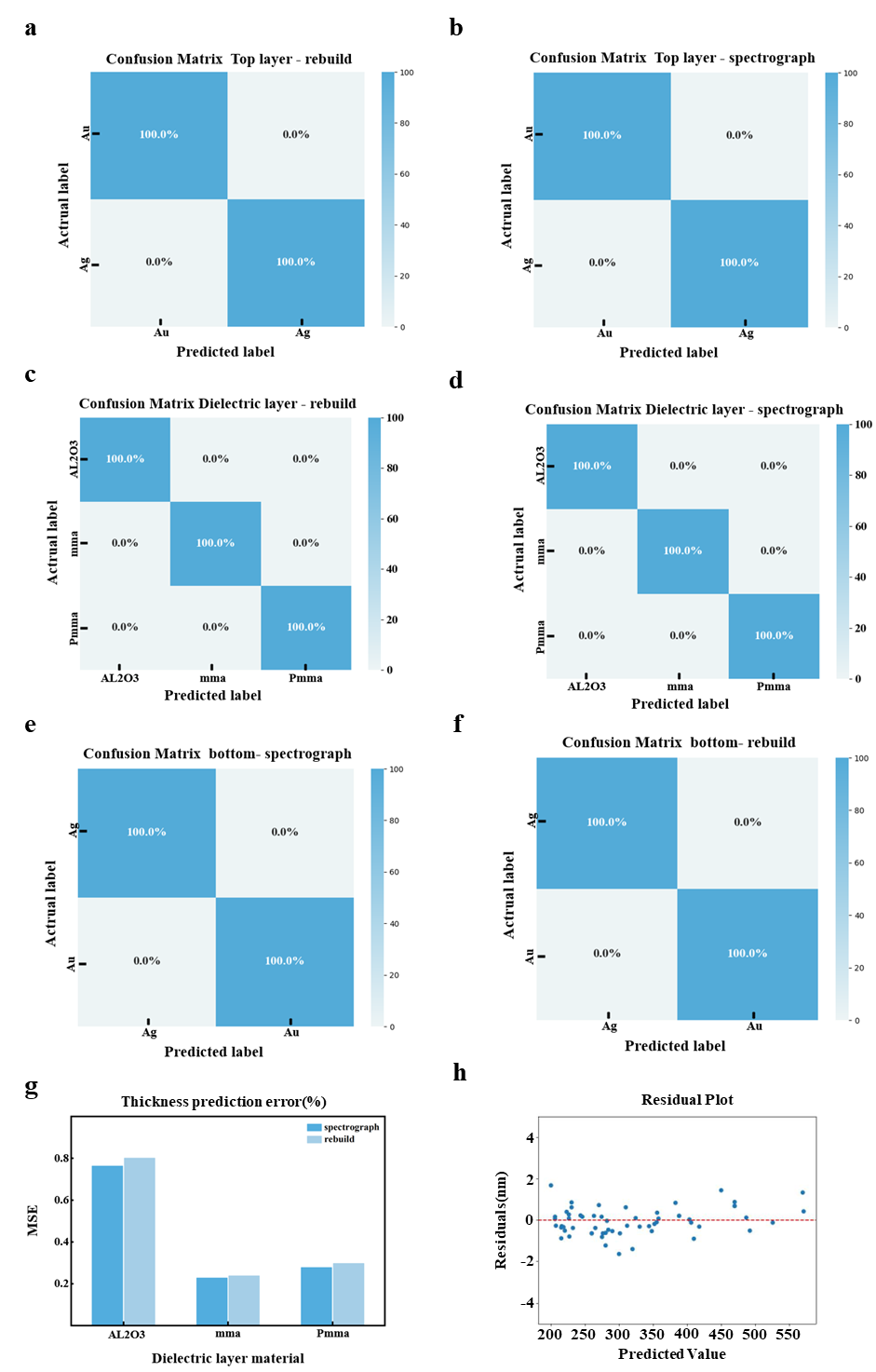


**Figure S10.** Statistical analysis of prediction errors for FP cavities in DNN networks. (a) The top-level confusion matrix for predicting the output of the sampled spectra from a microcomputing spectrometer. (b) Top level confusion matrix using traditional spectrometer sampling spectra as the output prediction. (c) Medium layer confusion matrix for predicting output spectra from MICS. (d) Medium layer confusion matrix using traditional spectrometer sampling spectra as output prediction. (e) The underlying confusion matrix for predicting the output of spectra sampled by MICS. (f) Bottom layer confusion matrix using traditional spectrometer sampling spectra as the output prediction. (g) MSE in thickness prediction. (h) Scatter plot of the error in thickness prediction.


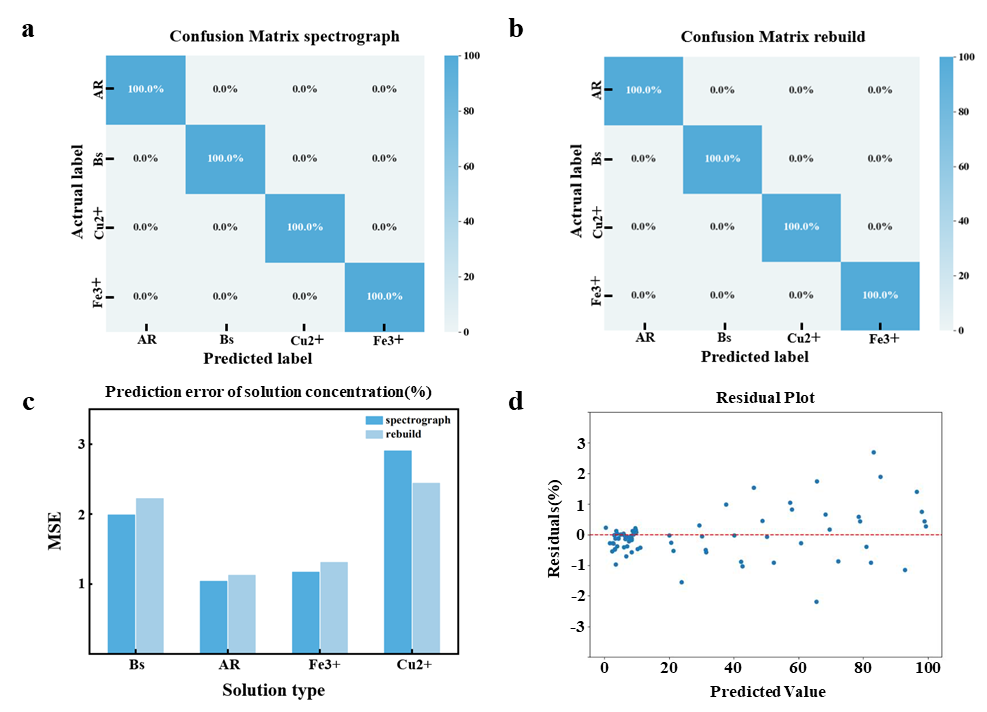


**Figure S11.** A statistical analysis of prediction errors in DNN network solutions. (a) The confusion matrix for solution solute prediction with MICS as the Output. (b) The confusion matrix for the prediction of the solution solute using a traditional spectrometer as the output. (c) MSE in predicting solution concentration. (d) Scatter plot of the error in predicting solution concentration.
